# Self-sustained planar intercalations due to mechanosignaling feedbacks lead to robust axis extension during morphogenesis

**DOI:** 10.1038/s41598-020-67413-8

**Published:** 2020-07-03

**Authors:** Samira Anbari, Javier Buceta

**Affiliations:** 1 Chemical and Biomolecular Engineering Department, Lehigh University, Bethlehem, 18015 USA; 2 Bioengineering Department, Lehigh University, Bethlehem, 18015 USA; 3 Institute for Integrative Systems Biology (I2SysBio), Consejo Superior de Investigaciones Científicas (CSIC)-Universitat de Valencia (UV), Parc Cientific, C/ Catedrático José Beltrán 2, 46980 Paterna, Valencia, Spain

**Keywords:** Computational models, Morphogenesis, Pattern formation, Computer modelling, Multicellular systems, Biological physics

## Abstract

Tissue elongation is a necessary process in metazoans to implement their body plans that is not fully understood. Here we propose a mechanism based on the interplay between cellular mechanics and primordia patterning that results in self-sustained planar intercalations. Thus, we show that a location-dependent modulation of the mechanical properties of cells leads to robust axis extension. To illustrate the plausibility of this mechanism, we test it against different patterning models by means of computer simulations of tissues where we implemented mechano-signaling feedbacks. Our results suggest that robust elongation relies on a trade-off between cellular and tissue strains that is orchestrated through the cleavage orientation. In the particular context of axis extension in Turing-patterned tissues, we report that different directional cell activities cooperate synergetically to achieve elongation. Altogether, our findings help to understand how the axis extension phenomenon emerges from the dynamics of individual cells.

## Introduction

During development, the initial spherical symmetry of the zygote undergoes complex changes in size and shape to form different tissues/organs and implement the body plan^1^. In that context, axis elongation, or more generically anisotropic growth, is a key morphogenetic geometric transformation that relies on the regulation of cellular activities due to the interplay between signaling and cell mechanical properties^2–8^. During axis extension, signaling events establish planar polarity patterns at the tissue level that feed back into the cellular dynamics, e.g., oriented mechanical responses. A notable example is convergent-extension (CE) due to cell intercalation events^9–12^. In other cases, a directed developmental expansion is achieved by translating polarity into differential growth events, oriented divisions, and/or active migration^13–15^. More recently, this problem has been addressed from the viewpoint of the physical (i.e., material-like) properties of tissues^16^. Recent relevant examples include the vertebrate body axis extension as a result of a jamming transition from a fluid-like to a solid-like behavior^17^. Yet, open questions remain. On the one hand, it is not clear how instructive signals arising from patterning are effectively translated into a non-equilibrium cellular dynamics that robustly sustains tissue extension and modulates the material-like properties spatiotemporally. On the other hand, further research is needed to understand how different mechanisms may contribute, cooperatively, to achieve anisotropic growth.

Some of these questions are beautifully illustrated during the outgrowth of the limb bud: a model system in morphogenesis to understand patterning and the directed developmental expansion of tissues^18,19^. In that regard, some models have suggested that tissue extension can be explained by a proliferation gradient hypothesis^20–23^. These models are supported by the demonstrated existence of a fibroblast growth factor (FGF) gradient that has its source at the apical ectodermal ridge (AER)^1^. However, while there is experimental evidence of a spatial modulation of the cell proliferation rates, it has been shown that this mechanism is not enough to generate a significant distal limb bud outgrowth^24^. Thus, it has been suggested that limb elongation must be driven by “other”, or additional, directional cell activities. Following these ideas, recent models based on the existence of an anisotropic filopodial-tension have proposed that limb outgrowth relies on a CE mechanism^25^. Notably, this model is able to resolve a conundrum observed during axis extension: cells’ elongation is perpendicular to the direction of the tissue elongation^24^. Still, how the pattern of gene expression observed in the limb bud modulates the cellular mechanics to generate such behavior in a sustained way is not understood.

Here we propose a framework to understand tissue elongation during morphogenesis, in particular that of the limb bud. Our model relies on the interplay between the mechanical properties of cells and the patterning that emerges from signaling events and provides positional information to the cells. Here we show that such a feedback, when combined with cellular growth and division, leads to auto-catalytic cell intercalations that can sustain tissue elongation robustly. We illustrate our proposal by means of numerical simulations of growing tissues using a vertex model approach^26,27^ that includes mechano-signaling feedbacks. We show the applicability of our proposal by means of two distinct patterning mechanisms: the French flag model^28^ and the Turing instability^29^.

## Results

### Auto-catalytic cell intercalation induces axis elongation in morphogen patterned tissues

**Fig. 1:**
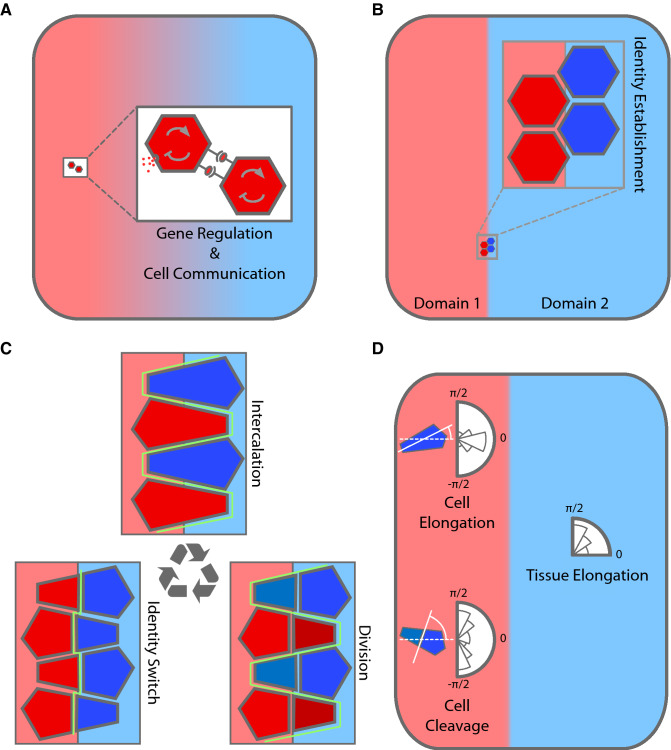
A mechanism to translate tissue planar polarity into self-sustained CE. Signaling and communication pattern the tissue (**A**) and establish positional information domains that provide cellular identity (**B**). (**C**) If cell identity implies distinct mechanical properties that promote cell intermingling, it leads to an auto-catalytic intercalation mechanism (see “Methods”). As schematically represented by the green line, cellular intercalation challenges the smoothness of boundary lines until cell identities are reassigned (“Methods”). (**D**) Directional features of cellular processes, such as elongation or cleavage, indicate that cells elongate along an axis perpendicular to the domain boundary, but the resulting CE arising for intercalations extends the tissue along a direction parallel to the domain boundary.

Our proposed auto-catalytic cell intercalation mechanism is schematically shown in Fig. 1 (“Methods”). We stress that cell intercalations that rely just on the differential adhesion hypothesis (DAH) cannot generate tissue elongation. It that case, distinct mechanical properties of cells (canonically promoting phase separation of homotypic populations^30–32^) are *inherited*. As a result, a transient CE may occur but, in the long term as cells grow and divide, it leads to isotropic tissue growth (Supplementary Fig. S1 and Supplementary Movie S1). Here, instead, we propose a modulation of the mechanical properties such that cellular *affinities* are assigned, dynamically, by positional information (instead of being inherited).

**Fig. 2:**
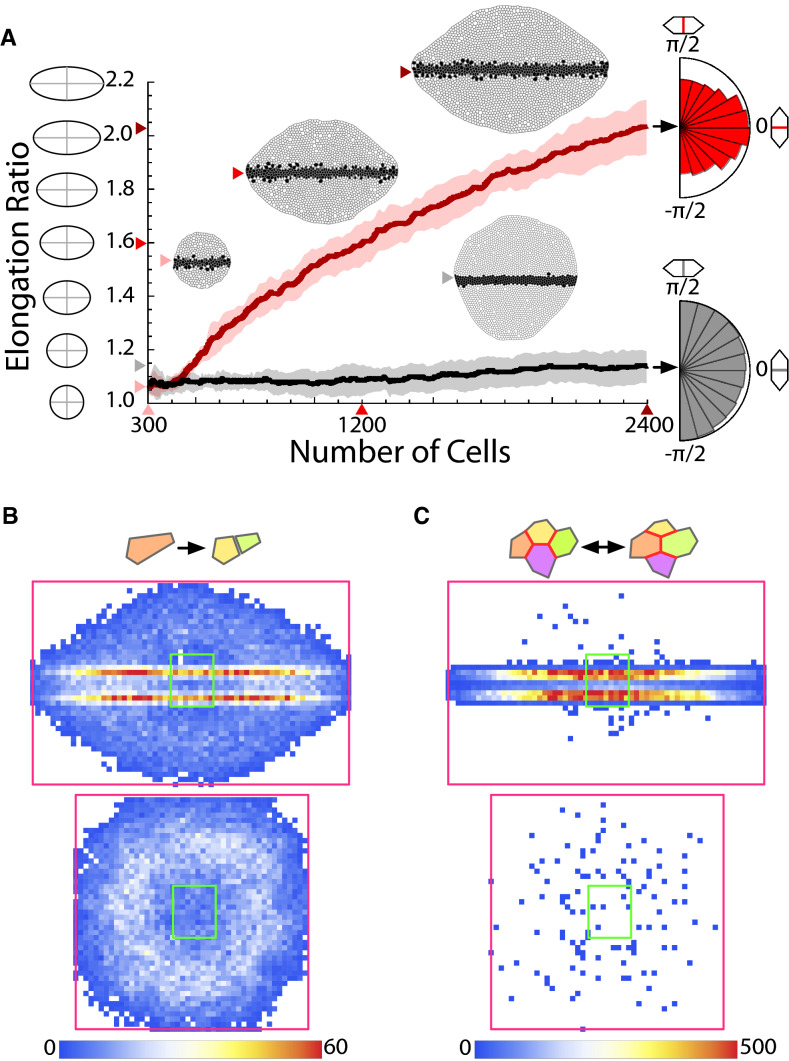
Coupling between tissue patterning and cell mechanics leads to robust elongation. (**A**) Elongation ratio as a function of the number of cells in a tissue patterned by a morphogen gradient (in silico experiments). Red: cell mechanics and patterning are coupled. Black: control simulations (adhesion not modulated by the pattern). Results from ten simulations: solid lines indicate the mean and the shading the standard deviation band. Values of average elongation, number of cells, and snapshots of representative simulations as indicated by the color arrows. The cumulative polar histograms of cleavage events (right) reveal that cells preferentially elongate perpendicular to the extension axis when the auto-catalytic intercalation mechanism applies. (**B**) Cumulative density histograms of divisions (all simulation frames and ten simulations). The green/magenta squares indicate the initial/final bounding boxes that delimit the tissue size. Intercalation-induced cell stretching (top) promotes cell divisions at the domain boundaries. (**C**) Cumulative density histograms of T1 transitions (all simulation frames and ten simulations). Auto-catalytic intercalation (top) provides fluidity to the tissue as revealed by the active remodeling at domain boundaries.

As a proof of concept to show its functionality in the context of tissue elongation, we simulated the case of a tissue patterned by a morphogen gradient (“Methods”)^33^. Our results show that a stationary gradient profile develops under tissue growing conditions (Supplementary Fig. S2 and Supplementary Movie S2). Cells acquire positional information by means of the French flag mechanism that sets domains of cellular identities depending on morphogen concentration thresholds (“Methods”)^28^. We assumed distinct cellular mechanical properties (adhesion, i.e., line tension in our vertex model implementation) as a function of the position of cells in the tissue. As a consequence, there was an increased “affinity” between cells from different domains (“Methods”). The latter promotes that cells with different identities located at domain boundaries intermingle. Figure 2A (Supplementary Movie S3) shows that the tissue elongates under these conditions. In contrast, elongation is not achieved in control simulations where the cell adhesion is not modulated by the morphogen signal (Supplementary Movie S4): for a precise mathematical definition of the elongation ratio see “Methods”. Figure 2A also confirms that the smoothness of domain boundaries is a proxy for the intercalation activity (“Methods”).

As for the role played by the orientation of cell divisions, in these in silico experiments, we implemented the Hertwig rule and included cell–cell variability as experimentally reported^34^ (“Methods”). Hence, the quantification of the cleavage orientation is as a proxy for the direction of the cellular elongation. Our results indicate that cells preferentially elongate perpendicular to the axis of extension of the tissue due to the intercalation events (Fig. 2A). If cell adhesion is not modulated by the morphogen signal we observed a randomized orientation of the cellular elongation as expected (Fig. 2A). Intercalation induces cell stretching that in turn promotes cell division as experimentally reported^35^. To test this possibility, we quantified the cumulative density of division events and found that cells indeed divided more actively at domain boundaries where intercalation is most operative (Fig. 2B). In contrast, when adhesion is not modulated by patterning, the positions where cell divisions occur are not correlated with thelocation of the domain boundaries. We also observed that inner cells divided less often due to the pressure increase^36^. Note also that, since our results revealed that intercalations promote cell divisions, we used the number of divisions as the timescale to quantify the duration of tissue growth in order to compare properly the elongation achieved through intercalation events and that of control simulations. Finally, to evaluate the plasticity (fluidity) of the tissue, we computed the cumulative density of T1 topological transitions (Fig. 2C). In that regard, we found that auto-catalytic intercalations largely increased the cellular activity, thus making the tissue more fluid.

### Robust elongation relies on a trade-off between cell and tissue stresses

**Fig. 3:**
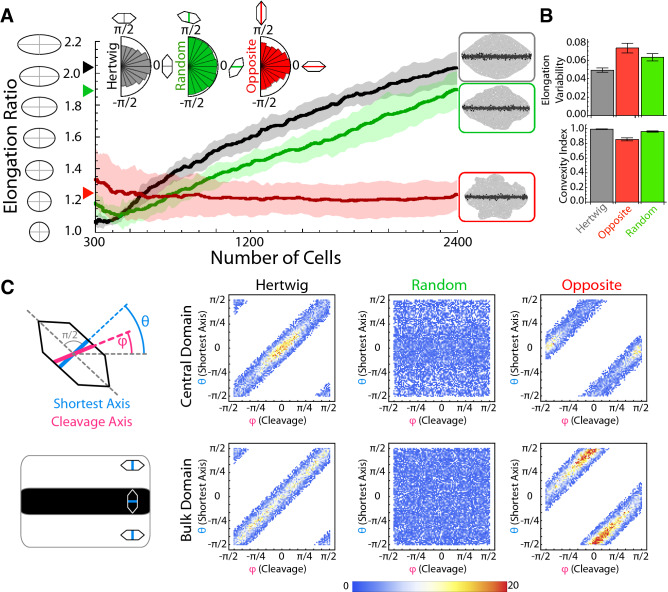
Effect of cleavage orientation. (**A**) Tissue elongation as a function of the number of cells for different cleavage dynamics (ten simulations). Solid lines stand for the mean and the shading for the standard deviation bands. The cumulative polar histograms of cleavage orientations (inset) are a readout of the cellular geometry when the Hertwig rule (black) or its opposite (red) apply but not in the case of random cleavage (green). The Hertwig rule leads to a systematic elongation. The insets show final snapshots of representative simulations depending on the cleavage dynamics. If cells do not divide perpendicularly to their longest axis, then the tissue develops irregularly. (**B**) The reproducibility of the elongation ratio and the irregularity of the tissue are quantified by the coefficient of variation (standard deviation/mean) of the elongation (last frame) and by the convexity index respectively (bars plot). (**C**) Analysis of the correlation (cumulative density histograms, all simulation frames and ten simulations) between the cellular geometry prior to division (quantified by the shortest cell axis angle, $\theta$) and the cleavage angle ($\varphi$) in different tissue domains.

In order to clarify more precisely the role played by oriented cell divisions during axis extension, we performed additional in silico experiments where we tested alternatives to the Hertwig rule: random orientation of the cleavage plane and divisions following the opposite of the Hertwig rule (cleavage plane parallel to the longest cell axis). As we did for the case of the Hertwig rule, we included variability (i.e., noise) in the cleavage orientation. We found that if the cleavage orientation followed the opposite of the Hertwig rule then the magnitude of the extension is lessened and the overall shape of the tissue is very irregular, Fig. 3A, B (Supplementary Movies S5 and S6). Random cleavage orientation implied an intermediate situation where elongation is achieved, but the tissue shape developed some irregularities (Fig. 3A, B). We quantitatively accounted for the tissue irregularity and reproducibility by computing the convexity index (“Methods”) and the coefficient of variation (variability) of the elongation (Fig. 3B). The results show that the Hertwig rule generates more regular tissues (convexity index closer to one) and more reproducible elongation (smaller coefficient of variation) than tissues subjected to random-orientation and opposite-Hertwig divisions.

Interestingly, in the case of the “opposite” rule, one would expect a cleavage statistics that would be the inverse to that found using the Hertwig rule. However, we found that cells having division planes parallel to the axis of extension are still predominant (polar histograms in Fig. 3A). To investigate this phenomenon, we examined the cleavage dynamics within the different domains of the tissue (Fig. 3C). Our results indicate that the auto-catalytic intercalation generates cellular stresses in the domain where this mechanism is more active (central domain) that contribute to elongate the cells perpendicularly to the extension axis. On the other hand, tissue elongation generates stresses in the cells of the “bulk” domain that promote their elongation along the axis of extension. This orthogonal orientation of the directions of the cellular elongation in different domains is, in fact, more clearly revealed when the “opposite” mechanism applies (Fig. 3C). Altogether, these results suggest that the interplay between cellular and tissue stresses, when coupled through oriented cell divisions (Hertwig rule) is instrumental to generate a robust axis elongation.

### Axis extension in turing patterned tissues depends on synergetic mechanisms

As shown above, the cell intercalation mechanism introduced herein implies an instructive role of signaling cues to establish the elongation axis: tissues extend along the direction of the domain boundaries determined by planar polarity. Thus, in primordia patterned following the French flag model the elongation axis is set by the signaling center where the morphogen is produced. This raises the question of whether the proposed mechanism applies to more complex patterning situations that need auxiliary mechanisms to establish planar polarity at the tissue level. To that end, we studied Turing patterned tissues. Developmental examples of the latter include animal coating^37^, the patterning of the tooth primordium^38^, the setting of the rugae spacing in the mammalian palate^39^, and a case that is particularly relevant in the context of tissue elongation: limb bud outgrowth^40,41^.

**Fig. 4:**
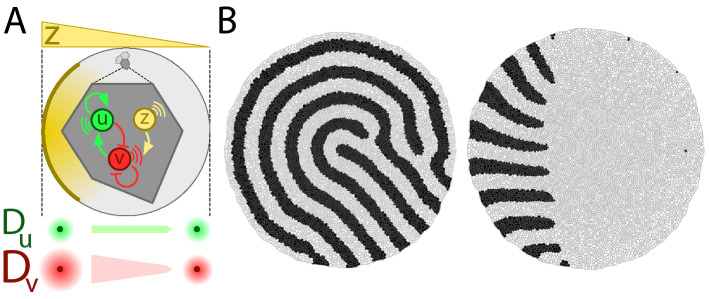
Stripe alignment mechanism in growing tissues. (**A**) In tissues where cells actively grow/divide, if each cell is driven by a regulatory network that just involves an activator, *u*, and an inhibitor, *v*, then the resulting Turing pattern displays rotational symmetry (panel **B** left). If an additional species, *z*, is released from the “tip” (left side of the tissue in this example) and set a polarity gradient such that the diffusivity of *v* is spatially modulated, then stripes align following the directionality of the gradient (panel **B** right). (**B**) Final snapshot of simulations without (left) and with (right) diffusivity modulation (constant cellular adhesion). The black (white) cellular domains account for regions where $u>v$ ($v>u$). Since diffusive transport relies on tissue topology (“Methods”) we avoided a possible bias in patterning by using in both simulations the same random sequences that determine the variability of cellular growth/division in order to reproduce the same cellular growth/division events and cell/tissue topologies.

Turing instabilities set distinct domains of expression in tissues. However, the patterns always display some level of rotational symmetry. Different ideas have been suggested to achieve stripe alignment (i.e., a symmetry-breaking event) in the context of Turing patterns^42^. In that regard, we found in our in silico experiments that, when tissues are subjected to cellular growth/division, the diffusivity-modulation mechanism due to the activity of a morphogen released from a cell population leads to pattern alignment consistently (Fig. 4, Supplementary Movies S7 and S8, “Methods”). We stress that the applicability of the auto-catalytic intercalation phenomenon is independent of the auxiliary mechanism that promotes stripe alignment. In the particular context of the limb bud, the FGF protein released from the AER would supposedly play the role of the morphogen that sets the planar polarity pattern that induces stripe alignment^40^. In addition, there is experimental evidence showing that FGF stimulates outgrowth and cellular proliferation^43^. Thus, we tested how axis extension in Turing patterned tissues depends on synergistic interactions between different mechanisms.

**Fig. 5:**
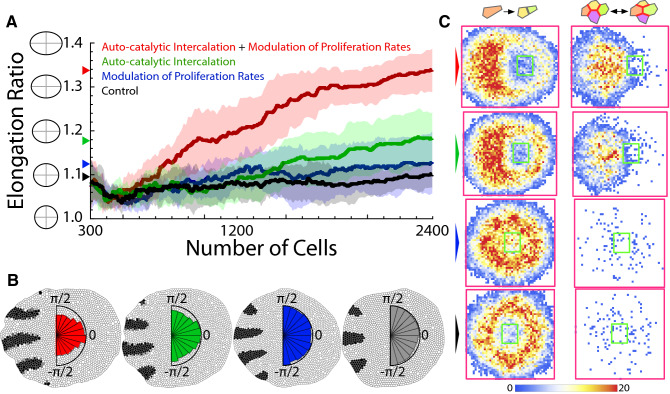
Elongation in Turing patterned tissues. (**A**) Comparison of tissue elongation as a function of the number of cells using different mechanisms (ten simulations): solid lines stand for the mean and the shading for the standard deviation bands. In all cases, cell cleavage follows the Hertwig rule. In control simulation, the mechanical properties of cells are independent of patterning (“Methods”). The final value of the average tissue elongation is indicated by the arrows. (**B**) Representative snapshots of simulations (same number of cells) and cumulative polar histograms of cleavage orientations (color codes and in **A**). The black (white) cellular domains account for regions where $u>v$ ($v>u$). When the auto-catalytic intercalation mechanism applies, cells elongate perpendicular to the direction of axis extension. (**C**) Cumulative density histograms (all simulation frames and ten simulations) of cell division events (left) and T1 transitions (right) depending on the elongation mechanism (color codes as in **A**). The green/magenta squares indicate the initial/final bounding box delimiting the tissue size.

Figure 5A (Supplementary Movie S9) shows that a combination of a spatial modulation of the cellular proliferation rates (cell cycle speed proportional to the morphogen signal released from the tip, “Methods”) and the auto-catalytic intercalation mechanism leads to a robust, and fast, axis extension. Motivated by prior studies that showed that a modulation of proliferation rates is not enough to generate a significant distal limb bud outgrowth^24^, we implemented in our simulations a very “mild” modulation. Figure 5A reveals that the modulation of proliferation rates alone leads to a $\sim 2\%$ increment of the elongation ratio with respect to control simulations (Supplementary Movie S10; control, Supplementary Movie S11). On the other hand, intercalation alone promotes tissue elongation (Fig. 5A and Supplementary Movie S12), but only when combined with the “mild” modulation of proliferation rates the elongation was boosted ($\sim 14\%$ increment). Also, as shown in Fig. 5B (polar histograms: divisions orientation), and in agreement with Fig. 3A, cells elongated, preferentially, perpendicular to the tissue expansion direction as long as the auto-catalytic intercalation mechanism applies and cell cleavage follows the Hertwig rule.

As in the case of the French flag model, we observed that division events are promoted in the intercalation region (Fig. 5C). Yet, we found a less structured (i.e., less digitate) distribution in agreement with limb bud outgrowth data^24^. As for the analysis of the topological remodeling of the tissue (T1 transitions), our data revealed a clear proximal-distal (right-left in the figure) gradient when the auto-catalytic intercalation mechanism applies such that there is more plasticity in the growing tip. We also observed a less structured T1 pattern in comparison with the French flag model simulations. The limited correlation between the location of division events and T1 transitions observed in the Turing model with respect to the results obtained in the morphogen simulations (cf. Figs. 2B, C, 5C, top) can be explained as follows. On the one hand, in the French flag model the signalling (central) domain is fixed in terms of its location and its width. Thus, the intercalation boundaries do not change their position noticeably (only grow their length). On the other hand, in the Turing case the pattern is highly dynamics and new domains, and hence intercalation boundaries, are created and disappearing as the tissue grows (e.g., Supplementary Movie S8). As a result, the cumulative statistics of division events and of T1 transitions are less correlated. Finally, as for the effect of the division dynamics we found, in agreement with the French flag model simulations, that either the random or the “opposite” cleavage dynamics contributed to a decrease of the reproducibility of the growth/elongation process with respect to the results obtained using the Hertwig rule: more irregularities and more variability as quantified by the convexity index and the coefficient of variation of the elongation rate respectively (Supplementary Fig. S3, Supplementary Movies S15–S18)]

## Discussion

Here we have proposed a framework to understand how the interplay between patterning and mechanics leads to axis extension. Our approach provides a simple and plausible mechanism that explains how the tissue-level planar polarity pattern feeds back to the cellular mechanics to produce sustained anisotropic growth elongation via auto-catalytic intercalations. This mechanism is based on some assumptions that can be justified by experimental observations of morphogenetic processes. First, cell identities are dynamically assigned depending on the location of the cells within a primordium following the positional information paradigm. Second, distinct identities imply distinct cell affinities (e.g., adhesion properties). Third, cell cleavage follows the Hertwig rule. Importantly, our results show that these premises lead to a non-equilibrium cellular dynamics able to explain the reported directional activities of cells during CE: intercalating cells elongate, predominantly, perpendicular to the direction of axis extension. Moreover, we have shown that the Hertwig rule is instrumental for the existence of a trade-off between cellular and tissues strains that contributes to a robust tissue elongation.

To illustrate the applicability of this mechanism we have used two patterning models: tissues patterned by morphogen gradients and tissues patterned by a Turing instability. Our simulations have been performed using a vertex model that implements a feedback between mechanical and signaling cues. Also, to check the robustness of our proposal, we have included different sources of variability: noise in the cleavage orientation and in the cell cycle duration. Our proposal does not aim at explaining in quantitative terms the elongation of a specific primordium but to show the plausibility of a generic mechanism. Still, we believe that our results are particularly relevant to understand the limb bud outgrowth. On the one hand, our findings are in qualitative agreement with the behavior found experimentally. On the other hand, we have shown, to the best of our knowledge for the first time, how the digitate pattern develops from a Turing instability using in silico experiments with a realistic cellular dynamics. An important implication of our results in the context of the limb bud is to reconcile data in terms of the possible mechanisms underlying the outgrowth. Thus, we have shown that the synergistic interaction between auto-catalytic intercalations and spatially modulated proliferation rates promotes elongation. Our data suggest that the former is the main driver of elongation and the latter boosts its effect (but it is not able to explain a systematic axis extension).

In our study, a “robust elongation” has been quantified in terms of the variability of the elongation achieved and also by assessing the regularity of the tissue shapes (convexity index). We point out that while natural variation in tissue development is essential for evolution, uncontrolled variation is detrimental to tissue function. These ideas are clearly exemplified by the bottleneck that organoids research is currently facing: the lack of reproducibility^44^. Here we have shown how patterns of gene expression and oriented cells divisions (Hertwig rule) cooperatively contribute to increase the similarity in phenotypic traits (size and shape). Consequently, our study sheds light into the mechanisms that challenge reproducibility and underlie pathological tissue growth.

We argue that our model could also provide insight into the recently reported fluidization during the vertebrate body axis elongation^17^. In that context, it has been shown that there is more tissue remodeling at the extending mesodermal progenitor zone and yet, the analysis of the orientation of neighbor exchanges revealed that no systematic alignment contributes to the elongation of the body axis. In that regard, here we have shown how patterning can promote gradients of tissue remodeling during elongation and, in fact, the directionality of neighbor exchanges is, counterintuitively, opposite to the extension direction. In that sense, our model could help to understand how primordia patterning affects the asymmetry of the tissue remodeling activity.

As a matter of discussion, here we have assumed that all the mechanical and biological interactions are described adequately by a 2D model in a planar geometry. This over-simplification is standard in the field and can possibly provide a plausible, yet basic, understanding of tissue remodeling. However, recent discoveries about the cellular behavior in 3D environments when tissues are subjected to some level of curvature, point towards an intriguing and important role of spatial T1 transitions^45^. Consequently, to investigate how the auto-catalytic intercalation model can the extended to include a realistic description of the 3D shape of the cells is an interesting subject for further studies.

In conclusion, we have presented a model based on hypotheses that seemingly connects the ideas of primordia patterning due to gene activity with oriented cellular activities in order to generate directional, yet robust, tissue growth. Therefore, our study paves the way to understand better shape remodeling and reproducibility during morphogenesis.

## Methods

### From cell signaling to tissue elongation: an auto-catalytic cell intercalation mechanism

Our proposed mechanism for autocatalytic cell intercalation is shown in Fig. 1.

Gene regulation and long/short-range cell-cell communication lead to a planar polarity pattern (Fig. 1A). Downstream signals further refine the pattern and provide positional information to cells in terms of different domains that determine cellular identities (Fig. 1B). If cell identity confers distinct mechanical properties that promote intermingling among cells from different domains, then cells in the neighborhood of domain boundaries intercalate to minimize their energy (Fig. 1C). As for the cellular division process, cell growth and intercalation-induced stretching coupled to the Hertwig rule (cleavage orientation perpendicular to the longest cell axis) set the preferential orientation of cleavage planes: parallel to domain boundaries^46,47^. Such bias in terms of the elongation and division orientation has been experimentally reported in a number of developmental processes including limb development^24,48–50^. Following division, the identities, and hence the mechanical properties, of daughter cells are reassigned depending on their position within the tissue. Dynamical assignment of cellular identities depending on their locations in a morphogenetic field is common during development, e.g.^51^. In that regard, experimental evidence about the dynamic establishment of cellular identities in the case of the limb bud primordium comes from micromass cultures where it has been shown that up-regulation, or down-regulation, of the skeletal marker *Sox9* depends on the relative positions of cells within the tissue^40^. This feedback between intercalation, division, and dynamic identity switching results in an auto-catalytic cell intercalation mechanism at the domain boundaries that leads to a self-sustained CE process (Fig. 1C). We notice that the intercalation-induced elongation relies on a clear separation of timescales: the cell-cycle is way larger than the timescale associated with mechanical effects as experimentally reported^52^. As schematically shown in Fig. 1C, we expect that the “smoothness” of the cell-identity boundaries is challenged by intercalation events until an identity switch occurs. Self-sustained intercalations cause tissue extension while cells elongate perpendicularly to the extension axis as represented by the cartoons of the polar histogram in Fig. 1D. See below for details about the mathematical formalization and implementation of this mechanism.

### Tissue simulations

Our approach is based on the vertex model originally developed by Nagai et al.^53^, and further adopted to model epithelial tissues by other authors, e.g.^52,54^. The model takes into account three energetic contributions for each cell vertex *i*:

1 $$\begin{aligned} E_{i}\left( t \right) =\sum \limits _{\alpha } \left[ \frac{K_{\alpha }}{2} \left( A_{\alpha }-A_{\alpha }^0 (t) \right) ^2+\frac{\Gamma _{\alpha }}{2}L_{\alpha }^2 \right] +\sum \limits _{\langle ij \rangle }\Lambda _{ij}l_{ij} \end{aligned}$$

index $\alpha$ corresponds to a cell, while *i* and *j* represent adjacent vertices sharing a connecting edge. The first term (r.h.s.) stands for the elastic energy of cells caused by the difference between the *actual* cell area $A_{\alpha }$ and the *preferred* cell area $A_{\alpha }^0$ (the area that the cell would like to have due to the cytoskeleton structure in the absence of the stresses associated with the adhesion and cortical tension). The second term, proportional to the squared cell perimeter, $L_{\alpha }$, describes the mechanical tension due to the elastic contraction of an actomyosin cortical ring. Finally, the third term describes the adhesion energy: $\Lambda _{ij}$ being a line tension coefficient (that can be either positive or negative) that weights the interaction between two cells, and where $l_{ij}$ represents the length of the edge connecting neighboring vertices, *i* and *j*. Based on this model, the cell packing geometries are determined by minimizing the total energy of the system which leads to a mechanical force balance where $\mathbf {F}_{i}=-\nabla E_{i}$. Under the assumption that inertia is negligible, the dynamics of cell vertices satisfies the equation of motion, $\eta \frac{d\mathbf {r}_{i}}{dt}=\mathbf {F}_{i}$ ($\eta$ being a drag/viscous coefficient). See^54,55^ for additional details.

In our simulations we used the following dimensionless parameter values $K=1$ and $\Gamma =0.02$ (all simulations), $\Lambda =\pm 0.05$ (simulations of DAH mechanism, Supplementary Fig. S1), $\Lambda =0.05$ (simulations of the morphogen gradient profile, Supplementary Fig. S2), $\Lambda =0.1$ (Turing stripe alignment formation, Fig. 4B). For all other simulations, $\Lambda$ ranges between 0 and 0.1 depending on the tissue domain see implementation of the signalling-mechanics feedback below (auto-catalytic cell intercalation). We imposed a value for the line tension for cell edges facing the tissue exterior of $\Lambda =0.2$. The latter promotes a circular shape of the tissue and helps to highlight that elongation or tissue deformation is due to the cellular dynamics and not to other effects.

As for the implementation of the cell cycle and the cellular growth, the cell cycle duration, $\tau$ , is a stochastic variable that satisfies $\tau =\epsilon t_{det} + \left( 1-\epsilon \right) t_{sto}$ where $t_{det}$ is a deterministic time scale that accounts for the average cell-cycle duration in the absence of mechanical stress and $t_{sto}$ is a random variable exponentially distributed with a probability density $\rho \left( t_{sto} \right) = \frac{\exp \left( -\frac{t_{sto}}{t_{det}} \right) }{t_{det}}$. The parameter $\epsilon$ weights the stochasticity of the cell-cycle duration (0.8 in our simulations). In our simulations $\langle \tau \rangle =1.5 \times 10^3$ (dimensionless). For the case shown in Fig. 4B $\langle \tau \rangle =7.5 \times 10^3$. If a proliferation gradient applies due to signaling (e.g., FGF), we simulated this effect by modulating the cell cycle duration $\langle \tau \rangle$ by the morphogen concentration (see details below). Cellular growth is implemented using a piece-wise dynamics that prescribes the following growth of the (dimensionless) preferred apical cell area, $A_{\alpha }^0\left( t\right)$: cells are quiescent up to the middle of their cell-cycle and then $A_{\alpha }^0\left( t\right)$ grows linearly (towards doubling) (see^54^ for details). With respect to the cleavage orientation, the code evaluates the inertia tensor of cells with respect to its centre of mass assuming that a proper representation of the former is a polygonal set of rods, i.e., the cell edges. The principal inertia axes indicate the symmetry axes of the cell: the longest axis of the cell is orthogonal to the largest principal inertia axis. Cells that divide following the Hertwig rule set their cleavage plane perpendicular to the longest cell axis. In simulations where cells divide opposite to the Hertwig rule or randomly, the cleavage plane is respectively parallel to the longest cell axis or random. Once the the *putative* division angle, $\varphi$, has been set, we implement variability by using a normal distribution $N \left( \varphi , \sigma ^2 \right)$. In our simulations $\sigma =0.2$ and we set bounds to the tails of the normal distribution such that the *actual* cell division lies within the interval $\left[ \varphi -0.5 , \varphi +0.5 \right]$. Cleavage is assumed to be instantaneous in our simulations.

As for the protein dynamics, we assume that cells are well-stirred systems where spatial effects can be disregarded. Each cell may contain a number of species (proteins) with dynamics described by a deterministic differential equation (see below). Protein numbers in each cell, are calculated by means of the Euler algorithm and protein concentration are obtained by dividing by the value of the cell area at a given time. Following a division event, proteins are distributed binomially between daughter cells. As for the diffusion of morphogen molecules, the diffusion operator is discretized in our simulations according to the cellular topology following^56^.

We simulate the tissue dynamics for $\sim 5$ cell cycles, yet defining two different temporal stages. First, starting with tissues that initially contain $10^2$ cells arranged in a regular hexagonal configuration, we “randomize” the tissue topology by cell growth and cleavage events and pre-pattern the tissue. During this simulation stage we do not consider any modulation of the mechanical properties due to signaling. This stage lasts $\sim 1.5$ cell cycles until the total number of cells in the tissue is $\sim 300$. After this transient, a second simulation stage is implemented during ~ 3.5–4 cell cycles until the total number of cells is $\sim 2.5\times 10^{3}$. During this stage, modulation of mechanical properties by signaling applies. All reported properties, e.g., elongation ratio, are calculated only during the second simulation stage.

### French flag patterning model

We implemented the French flag patterning scheme by simulating, first, a signaling center from which a morphogen, *c*, diffuses out. The dynamics of the morphogen concentration for a cell *i*, $c_{i}$, is prescribed following^57^:

2 $$\begin{aligned} \frac{\partial c_{i}}{\partial t}=D_{c}\nabla ^2c_{i}-k_{c}c_{i}+2j_{c} \mathcal {H} \left( y_{i}-y_{a} \right) \mathcal {H} \left( y_{b} - y_{i} \right) , \end{aligned}$$

where $y_{i}$ stands for the vertical coordinate of the geometrical center of cell *i*, $\mathcal {H} \left( z \right)$ is the Heaviside step function, $D_{c}$ is the diffusion coefficient, $k_{c}$ the degration rate, and $j_{c}$ the morphogen current: $-D_{c}\partial c_{i}\partial y = j_{c}$ in the domain $y_{i}\in \left( y_{a},y_{b}\right)$. Thus, the morphogen is released from all cells with centers in the range $\left( y_{a},y_{b}\right)$. In our simulation the parameter values are (dimensionless): $D_{c}=10^{-2}$, $k_{c}=2.54\times 10^{-3}$, $j_{c}=398$, and $\left( y_{a}, y_{b}\right) =\left( 3.5,5\right)$. Taking into account that $A_{\alpha }^0\in \sim \left( 1-2\right)$, the width of the signaling center typically comprises ~ 1–2 cells. Given the Eq. (2), if $\Delta y=y_{b}-y_{a}\rightarrow 0$ then the stationary concentration of the morphogen in a cell at a location $y_{i}$ reads $c_{i}=c_{0}e^{-\frac{y_{i}}{\lambda }}$ with $c_{0}\sim 10^5$ and $\lambda =\sqrt{\frac{D}{k}}\simeq 2$ being the typical decay length of the morphogen^57^. To implement a French flag positional information mechanism, we set a morphogen threshold of $c_{t}=3.5\times 10^4$ molecules/cell and defined the following rate dynamics of two putative proteins, $d_{1}$ and $d_{2}$ for every cell, *i*,

3 $$\begin{aligned} \frac{\partial d_{1,i}}{ \partial t}= & {} \mathcal {H} \left( c_{i}-c_{t} \right) - d_{1,i}, \end{aligned}$$

4 $$\begin{aligned} \frac{\partial d_{2,i}}{ \partial t}= & {} \mathcal {H} \left( c_{t}-c_{i} \right) - d_{2,i}. \end{aligned}$$

Thus, cell identities and tissue domains are characterized by a vectorial tag: central domain cells $\left( d_1,d_2\right) =\left( 1,0\right)$, bulk domain cells $\left( d_1,d_2\right) =\left( 0,1\right)$. Taking into account the value of $c_{t}$ and the parameter used, the central domain has a typical width of 4–5 cells.

### Turing patterning model

In our simulations we used a generic reaction–diffusion model that can be mapped into an activator-substrate model that has been proposed to describe pigmentation patterns^58^ or into an activator-inhibitor model to describe regeneration^59^. More recently the model has been used to explored the role of the so-called protein *granular* noise due to discretization effects during patterning^60^. The model describes the concentration of two proteins, *u* and *v*, in every cell *i*, that can undergo a Turing instability leading to labyrinth-like patterns with rotational symmetry:

5 $$\begin{aligned} \frac{\partial u_{i}}{ \partial t}= & {} a\left[ \left( u_{i}-u_{0}\right) +\left( v_{i} - v_{0} \right) \right] -\frac{\left( u_{i}-u_{0} \right) ^3}{2}+ \nabla ^2 u_{i}, \end{aligned}$$

6 $$\begin{aligned} \frac{\partial v_{i}}{ \partial t}= & {} -2\left( u_{i}-u_{0}\right) -\left( v_{i}-v_{0}\right) + D_{v}\nabla ^2 v_{i}. \end{aligned}$$

In our simulations we used the dimensionless parameters of $a=0.9$ for all simulations and $D_{v}=9$ for the simulations shown in Fig. 4B which lead to patterns around the homogeneous state $u_{0}=v_{0}=2$. For details about the Turing instability condition and non-linear effects in this model see^61^.

Stripe alignment was obtained by implementing an anisotropic diffusion mechanism of species *v*. To do so, we defined a cell population with identity $\mathcal {I}=Z$ at the boundary of the tissue (see Fig. 4A) that produces a morphogen, *z* (see Supplementary Videos S9–S10),

7 $$\begin{aligned} \frac{\partial z_{i}}{\partial t}=D_{z}\nabla ^2 z_{i}-k_{z} z_{i}+2j_{z} \delta _{\mathcal {I}_{i},Z}. \end{aligned}$$

The parameter values (dimensionless) used in our simulations were: $D_{z}=0.75$ ($D_{z}=1.5$ in Fig. 4B), $k_{z}=1.25\times 10^{-2}$, $j_{z}=5\times 10^{-2}$. Under those conditions $z\sim 1$ at locations where $\mathcal {I}=Z$. Protein *z* modulated the diffusivity of protein *v* linearly such that $D_{v_{i}}=A z_{i}+B$ with $A=43.3$ (Fig. 4B, right), $A=13$ (simulations about tissue elongation), and $B=4$ in all cases.

Similarly to the case of morphogen patterned tissues, we defined additional putative proteins to provide identities to cells,

8 $$\begin{aligned} \frac{\partial d_{1,i}}{ \partial t}= & {} \mathcal {H} \left( (u_{i}-u_{0})-(v_{i}-v_{0})-\Delta \right) -d_{1,i}, \end{aligned}$$

9 $$\begin{aligned} \frac{\partial d_{2,i}}{ \partial t}= & {} \mathcal {H} \left( \Delta -(u_{i}-u_{0})-(v_{i}-v_{0})\right) - d_{2,i}, \end{aligned}$$

where $\Delta =0.2$ is a concentration threshold. That is, if $(u-u_{0})-(v-v_{0})>\Delta$, cells are characterized by a vectorial tag $\left( d_1,d_2\right) =\left( 1,0\right)$ and if $(u-u_{0})-(v-v_{0})< \Delta$ then $\left( d_1,d_2\right) =\left( 1,0\right)$. Since the characteristic domain size as a function of the pattern wavelength, $l_{c}$, is $l_{c}/2$, and taking into account that (see^61^),

10 $$\begin{aligned} l_{c}=\frac{2\pi \sqrt{2}}{\sqrt{a-\left( 1/D_{v}\right) }}=\frac{2\pi \sqrt{2}}{\sqrt{a-\left( 1/\left( Az+B\right) \right) }} \end{aligned}$$

then the domains at locations where $\mathcal {I}=Z$, i.e., $z_{i}\simeq 1$, comprise $\sim$ 5–6 cells (Fig. 4). The patterning disappear at locations where $D_v=\left( 3+2\sqrt{2}\right) /a$^61^.

The *z*-morphogen concentration profile is further used to generate a proliferation gradient in some simulations (see text). In that case the average cell cycle duration as a function of *z* is $\langle \tau \rangle _{i}=\frac{\hat{A}}{\hat{A} z_{i}+\hat{B}} \cdot \langle \tau \rangle$ with $\langle \tau \rangle =1.5 \times 10^3$, $\hat{A}=4$, and $\hat{B}=2$. The cycle duration then varies from $10^3$ ( locations where $z\sim 1$) to $3\cdot 10^3$ (locations where where $z\sim 0$).

### Auto-catalytic cell intercalation

The patterning-mechanics interaction is implemented in our model through the putative proteins $d_1$ and $d_2$ that characterize, dynamically, the positional information depending on the underlying gene regulation. Thus, the following matrix describes the identity relation between a pair of cells neighboring *i* and *j*,

11 $$\begin{aligned} \mathcal {I}_{\langle i,j\rangle }=\left( \begin{matrix} d_{1,i} &{} d_{2,i} \\ d_{1,j} &{} d_{2,j} \end{matrix} \right) . \end{aligned}$$

Consequently, if cells *i* and *j* belong to the same positional information domain then $\left| \mathrm {det}\left( \mathcal {I}_{\langle i,j\rangle }\right) \right| =0$ and if cells *i* and *j* belong to different positional information domain then $\left| \mathrm {det}\left( \mathcal {I}_{\langle i,j\rangle }\right) \right| =1$. In our simulations we modulated the adhesion energy between two neighboring cells by means of the following dependence of the line tension parameter, $\Lambda _{i,j}$, as a function of $\mathcal {I}_{\langle i,j\rangle }$, see Eq. (1): $\Lambda _{ij}=\Lambda _{0}+\gamma \left| \mathrm {det}\left( \mathcal {I}_{\langle i,j\rangle }\right) \right|$ with $\Lambda _{0}=-\gamma =10^{-1}$. As a consequence, cell intercalation is promoted at domain boundaries.

### Tissue elongation ratio

The tissue elongation ratio is computed as follows. We first estimate the center of mass of the tissue using the perimetric cell vertices. Second, we calculate the components of the inertia tensor with respect the center of mass of the tissue:

12 $$\begin{aligned} I_{jk}= \sum \limits _{i} \left( r_{i}^2 \delta _{jk} - x_{i,j} x_{i,k} \right) , \end{aligned}$$

where the sum runs for all the perimetric vertices, *i*, with Cartesian coordinates $\left( x_{i}, y_{i}\right) =\left( x_{i,1}, x_{i,2}\right)$, $r_{i}$ is the distance to the center of mass, and $\delta _{jk}$ is the Kronecker delta. Finally, we obtained the tissue elongation ratio by calculating the ratio of the two eigenvalues of the inertia tensor.

### Growth reproducibility: convexity index and elongation variability

We characterize the shape irregularity of tissues by means of the convexity index^62^:

13 $$\begin{aligned} \mathcal {C}=\frac{\text {convex perimeter}}{\text {perimeter}} \end{aligned}$$

using the perimetric cell vertices. Thus, in tissues with no growth irregularities (e.g., overhangs, finger-like structures) $\mathcal {C}\sim 1$. The reproducibility of the results obtained in different simulations is evaluated by computing the coefficient of variation (ratio of the standard deviation over the mean) of the elongation ratio in the last final frame of our simulations.

### Cells division and T1 transitions

The location of cell divisions is computed by collecting the coordinates of the centers of mother-cell right before cleavage. As for T1 transitions, we registered the coordinates of the edge associated to neighbor exchanges before, $l^{b}_{i,j}$, and after, $l^{a}_{i,j}$, a transition. The location of a T1 transition is characterized by the intersection point of the edges $l^{b}_{i,j}$ and $l^{a}_{i,j}$.

## Supplementary information

Supplementary file1 (ZIP 366485 kb)
