# Supplementary material for: Self-sustained planar intercalations due to mechanosignaling feedbacks lead to robust axis extension during morphogenesis
Source: Sci Rep. 2020 Jul 3;10:10973. doi: 10.1038/s41598-020-67413-8 (PMC7334228; doi:10.1038/s41598-020-67413-8)
Supplement: Supplementary file 1 — Supplementary file1 (ZIP 366485 kb) [file 41598_2020_67413_MOESM1_ESM.zip › supplementarymaterial.pdf]

Self-sustained Planar Intercalations due to Mechanosignaling Feedbacks Lead to Robust Axis Extension during Morphogenesis
(*Supplementary Material*)

Samira Anbari[1] and Javier Buceta[1,2,*]
[1] *Chemical and Biomolecular Engineering Department, Lehigh University, Bethlehem 18015, USA and*
[2] *Bioengineering Department, Lehigh University, Bethlehem 18015, USA*
[*] *Corresponding author: jbuceta@lehigh.edu*

SIMULATION CODE: COMPILATION INSTRUCTIONS

Data and simulation codes for generating each of the figures and movies of the study are provided within the folder `CODE`. The `SRC` folders contain the required C++ source files to generate an executable code when compiled with a standard C++ compiler. In our case the code was compiled in a Linux system using g++. The name of the folders indicates the corresponding figure and/or movie in the main or supplementary text. The executable file is generate through a `Makefile` (i.e., invoking the `make` command within the directory containing the source code).

SIMULATION CODE: DATA FILES OUTPUT

As a result of a particular simulation different output files are generated. For each simulation, the relevant output files are collected in the `DATA` folder. The output file `dcells.dat` contains the information of all tissue cells for every frame registered in the simulation. The structure of each frame is as follows:

```
#cells stageidx
cellID type #vertexes area #proteinspecies #protein1 #protein2 ...  #proteinN centerx centery
idxneighbor1 idxneighbor2 ...  idxneighborN vertex1x vertex1y vertex2x vertex2y ...  vertexNx vertexNy
cellID type #vertexes area #proteinspecies #protein1 #protein2 ...  #proteinN centerx centery
idxneighbor1 idxneighbor2 ...  idxneighborN vertex1x vertex1y vertex2x vertex2y ...  vertexNx vertexNy
.
.
.
cellID type #vertexes area #proteinspecies #protein1 #protein2 ...  #proteinN centerx centery
idxneighbor1 idxneighbor2 ...  idxneighborN vertex1x vertex1y vertex2x vertex2y ...  vertexNx vertexNy
```

That is, each frame starts with a single line that indicated the number of cells in that frame and the stage index. The former, in turn, indicates the number of lines in each frame. Each of those lines contains information of a cell in the following order: the ID of the cell $(0, 1, 2...)$, the type of the cell $(1, 2, 3, ...)$, the number of vertexes, the cell area, the number of protein species, their values (number of proteins), the coordinates of the cell center, the indexes of its neighbors, and the coordinates of the cell vertexes (clockwise orientation). For information about the number of protein species and their order for each simulation, see the `protein_order.dat` file that is generated when running the code.

The ID of the cell (`cellID`) is a unique string that is maintained up to a division event: following a division each daughter cell receives a new ID that is the result of joining (by means of hyphens) the ID of the mother plus and an additional number. Thus, the cell ID allows to reconstruct its lineage: e.g., the cell `405-345-33-67` is a third generation cell (number of hyphens) originating from cell `405` of the initial tissue (its grand-grandmother) and its mother and grandmother are cells `405-345-33` and `405-345` respectively. We point out that the index of a neighbor (e.g., `idxneighbor1`) does not correspond to a *real* ID of the cell (i.e., a string such as `405-345-33-67`) but to a number that is the internal ID of the cell in the code. The latter corresponds to the ordinal index of the line within the frame minus 1 (i.e., cell identities counting starts with 0). For example an index of the neighbor `564` corresponds to the cell that is the line `565` in the listing of cells in that frame. The code generates also an output file `divisions.dat` that accounts for the information about cell divisions. Each line of the file reads:

```
stageidx idxduration idxintermediate idxmothercell IDdaughter1 IDdaughter2 type divisionangle
divisionanglehertwig divisionangleplanned centerx centery area #cells #vertexes vertex1x vertex1y
vertex2x
vertex2y ...  vertexNx vertexNy
```

Each line indicates the stage index and those of an external loop (`duration`) and an internal loop (`intermediate`) that account for the time evolution of the simulation: each `duration` step contains a given number of `intermediate` steps such that the total (dimensionless) time of a simulation reads (`duration`$\times$`intermediate`$\times \Delta t$ (where Δt is the time step used in the Euler algorithm). Other information provided is the *internal* ID of the mother cell, the *real* IDs of daughter cells, their type, the *actual* division angle, `divisionangle`, the division angle assuming no deviation from the Hertwig rule, `divisionanglehertwig`, the planned division angle, `divisionangleplanned`, the coordinates of the cell center (mother cell), the *actual* cell area at division time (mother cell), the updated number of cells in the tissue, the number of vertexes (mother cell), and the coordinates of those.

Finally, the code generates a log file, `tifosi.log`, where the processes that change the topology of the tissue (e.g., a T1 transition) are recorded . The file keeps the time of the event by indicating the `stageidx`, the `idxduration`, and `idxintermediate` and additional information of the process. In the case of a T1 transition, it is provided the

internal ID of the edge that disappears, its coordinates (i.e., those of the vertexes that define the edge), the *real* IDs of the cells that shared the edge prior to the T1 transition, the coordinates of the new edges after the transition, and the real IDs of the cells that become connected after the transition takes place. An example of how a T1 transition is captured in the log file reads:

```
1 0 3733: t1 process on edge 738 (21.219, 12.1942)-(21.2691, 12.2308) that divides cells 245 and
225.
Coordinates after transition: (21.2623, 12.1874)-(21.2257, 12.2375) and divides cells 224 and 246.
```

In the case of a T2 transition (disappearing triangular cell), on top of registering the timing of the event, the log keeps a record of the *real* ID of the triangular cell, the internal ID of the edge that disappears the first (and its coordinates prior to the transition), and also the real IDs of the cell that shared that edge. For example,

```
1 2 7452: t2 process on cell 224 triggering edge 736 (19.3435, 14.0312)-(19.2848,14.0112)
that divides cells 225 and 224.
```

In the case of a T3 transition (two neighboring triangular cells that simultaneously disappear) the information is similar to that of a T2 transition and the log file reads:

```
1 23 45632: t3 process on cell 45-345-12 and cell 34 triggering edge 7 (1.3367, 20.4561)-(19.3490,
0.6511)
that divides cells 45-345-12 and 73-465.
```

Finally, in the case of a division event, the timing and real IDs of the daughter cells are recorded , for example,

```
1 74 8724: cell division. Daughter cells: 252-401 and 252-400
```

SUPPLEMENTARY VIDEOS

- Movie S1. The DAH mechanism cannot generate axis extension.

- Movie S2. Time evolution of a morphogen concentration gradient in the tissue where cells are actively growing and dividing.

- Movie S3. Simulation of the auto-catalytic intercalation mechanism in a tissue patterned by a morphogen gradient (Hertwig rule).

- Movie S4. Simulation of tissue growth if adhesion is not modulated by the morphogen signal (Hertwig rule).

- Movie S5. Simulation of tissue growth if adhesion is not modulated by the morphogen signal (opposite Hertwig rule).

- Movie S6. Simulation of tissue growth if adhesion is not modulated by the morphogen signal (random orientation of divisions).

- Movie S7. Simulation of Turing instability without diffusivity modulation.

- Movie S8. Simulation of Turing instability with diffusivity modulation..

- Movie S9. Simulation of tissue growth: Turing patterned tissue with auto-catalytic intercalations and modulation of cellular proliferation (Hertwig rule).

- Movie S10. Simulation of tissue growth: Turing patterned tissue without auto-catalytic intercalations and with modulation of cellular proliferation (Hertwig rule).

- Movie S11. Simulation of tissue growth: control simulations (Turing patterned tissue) without auto-catalytic intercalations or modulation of cellular proliferation (Hertwig rule).

- Movie S12. Simulation of tissue growth: Turing patterned tissue with auto-catalytic intercalations and without modulation of cellular proliferation (Hertwig rule).

- Movie S13. Cell population producing the morphogen responsible of stripe alignment and the modulation of cellular proliferation (Turing patterned tissue: auto-catalytic intercalations and modulation of cellular proliferation, Hertwig rule).

- Movie S14. Morphogen concentration profile driving the stripe alignment and the modulation of cellular proliferation (Turing patterned tissue: auto-catalytic intercalations and modulation of cellular proliferation, Hertwig rule).

- Movie S15. Simulation of tissue growth: Turing patterned tissue with auto-catalytic intercalations and modulation of cellular proliferation (opposite-Hertwig rule, example 1).

- Movie S16. Simulation of tissue growth: Turing patterned tissue with auto-catalytic intercalations and modulation of cellular proliferation (opposite-Hertwig rule, example 2).

- Movie S17. Simulation of tissue growth: Turing patterned tissue with auto-catalytic intercalations and modulation of cellular proliferation (random cleavage orientation, example 1).

- Movie S18. Simulation of tissue growth: Turing patterned tissue with auto-catalytic intercalations and modulation of cellular proliferation (random cleavage orientation, example 2).

SUPPLEMENTARY FIGURES

FIG. 1. **(Figure S1)** Starting from the same initial configuration, if the cell adhesion (line tension parameter) is the same for different cell populations, **A**, then cell intercalations no not occur . If the adhesion between cells promotes cell intermingling, **B**, cell intercalation is observed. However, the fact that the cell mechanical properties are inherited (instead of being dynamic) leads to isotropic tissue growth in the long term.

FIG. 2. **(Figure S2)** (**A**) Time evolution of a morphogen concentration gradient in a tissue where cells actively grow and divide. The profile reaches a stationary state, **B**.

FIG. 3. **(Figure S3) Effect of cleavage orientation in Turing patterned system. A**: Tissue elongation as a function of the number of cells for different cleavage dynamics (ten simulations): solid lines stand for the average value of the elongation and the shading for the standard deviation bands. The reproducibility of the elongation process and the irregularity of the tissue are quantified by the coefficient of variation (standard deviation/mean) of the elongation ratio (last frame) and by the convexity index respectively (bars plot). The Hertwig rule leads to the smallest variability indicating a more robust elongation and regular shape. **B**: The polar histograms of cleavage orientations (inset) are a readout of the cellular geometry when the Hertwig rule (black) or its opposite (red) apply but not in the case of random cleavage (green). Final snapshots of representative simulations depending on the cleavage dynamics. Two snapshots for opposite and random cleavage are shown. Those correspond to illustrative cases where the elongation ratio is smaller/larger than average. Deviations from the Hertwig rule leads to irregular tissue shapes (see quantification in **A**).
