# Supplementary figures and images for: Self-sustained planar intercalations due to mechanosignaling feedbacks lead to robust axis extension during morphogenesis
Source: Sci Rep. 2020 Jul 3;10:10973. doi: 10.1038/s41598-020-67413-8 (PMC7334228; doi:10.1038/s41598-020-67413-8)

# A

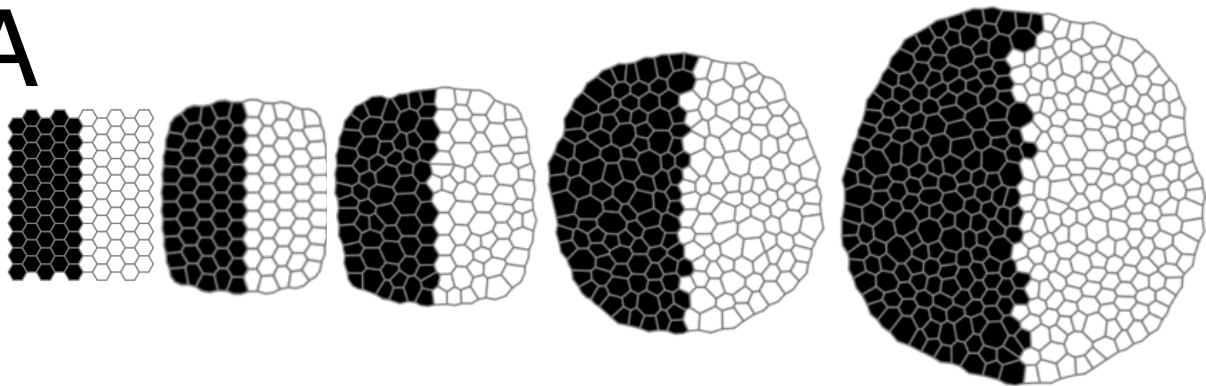

# B

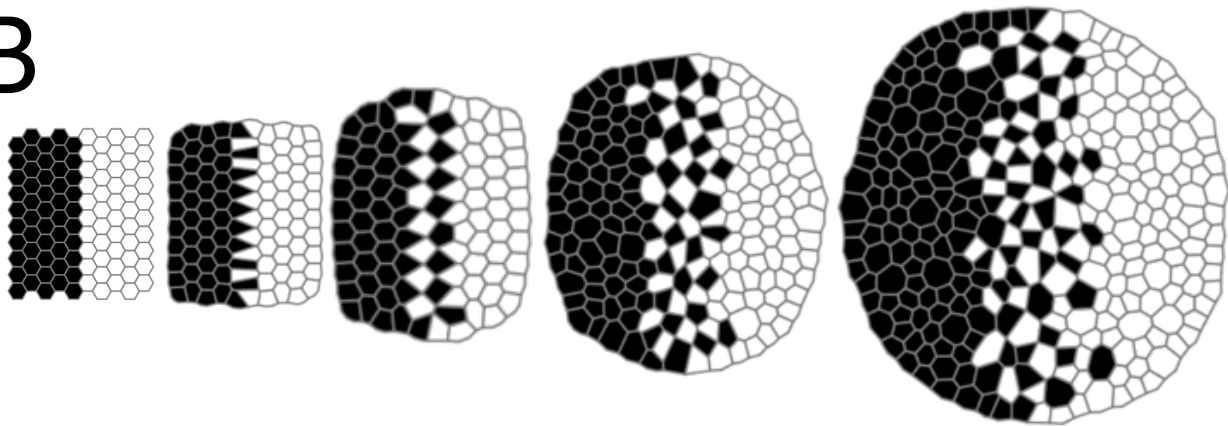

Supplement: Supplementary file 1 — Supplementary file1 (ZIP 366485 kb) [file 41598_2020_67413_MOESM1_ESM.zip › FigS1.pdf]

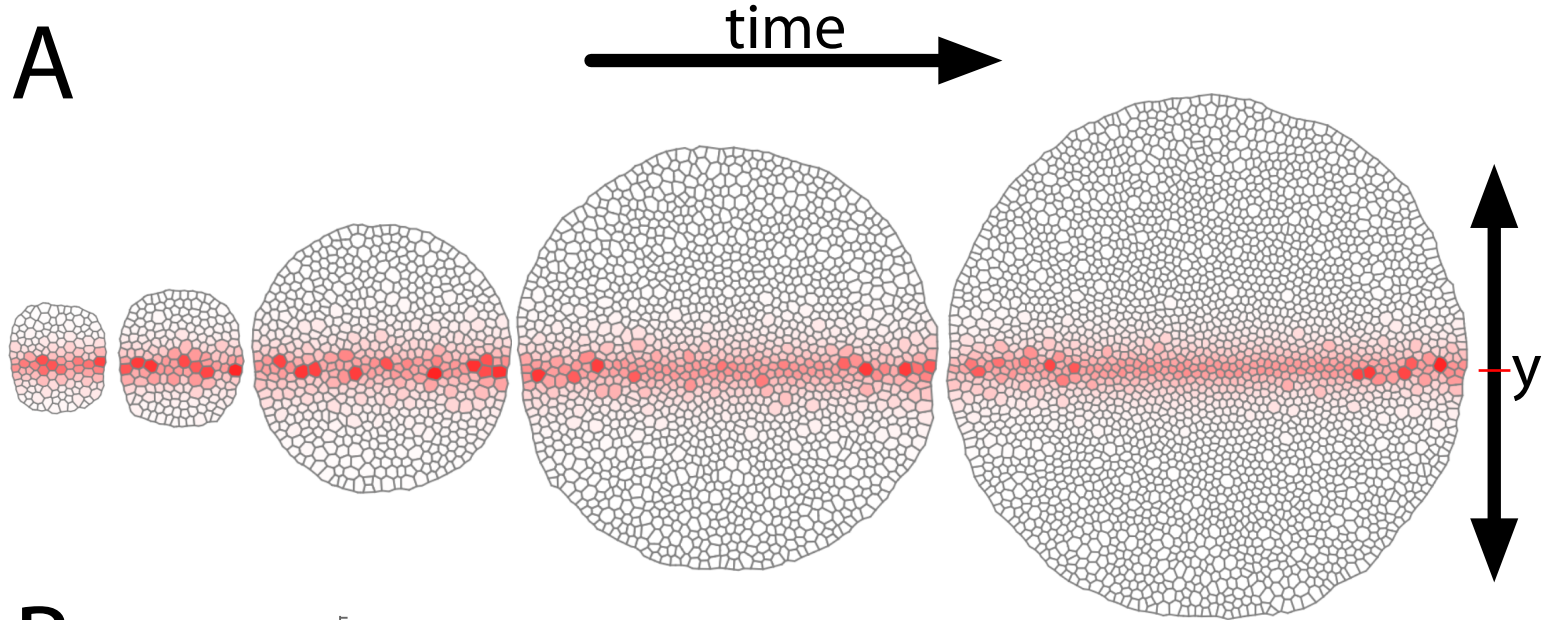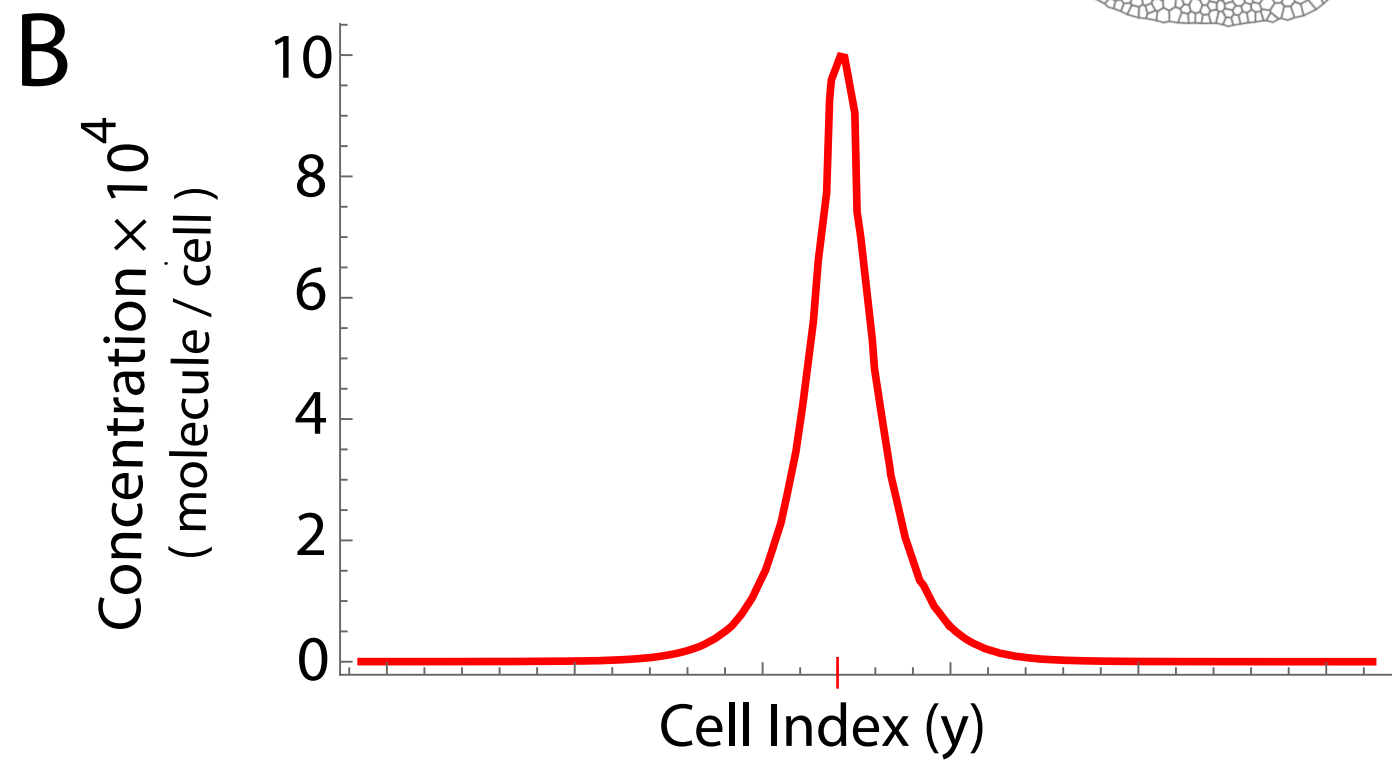

Supplement: Supplementary file 1 — Supplementary file1 (ZIP 366485 kb) [file 41598_2020_67413_MOESM1_ESM.zip › FigS2.pdf]

A

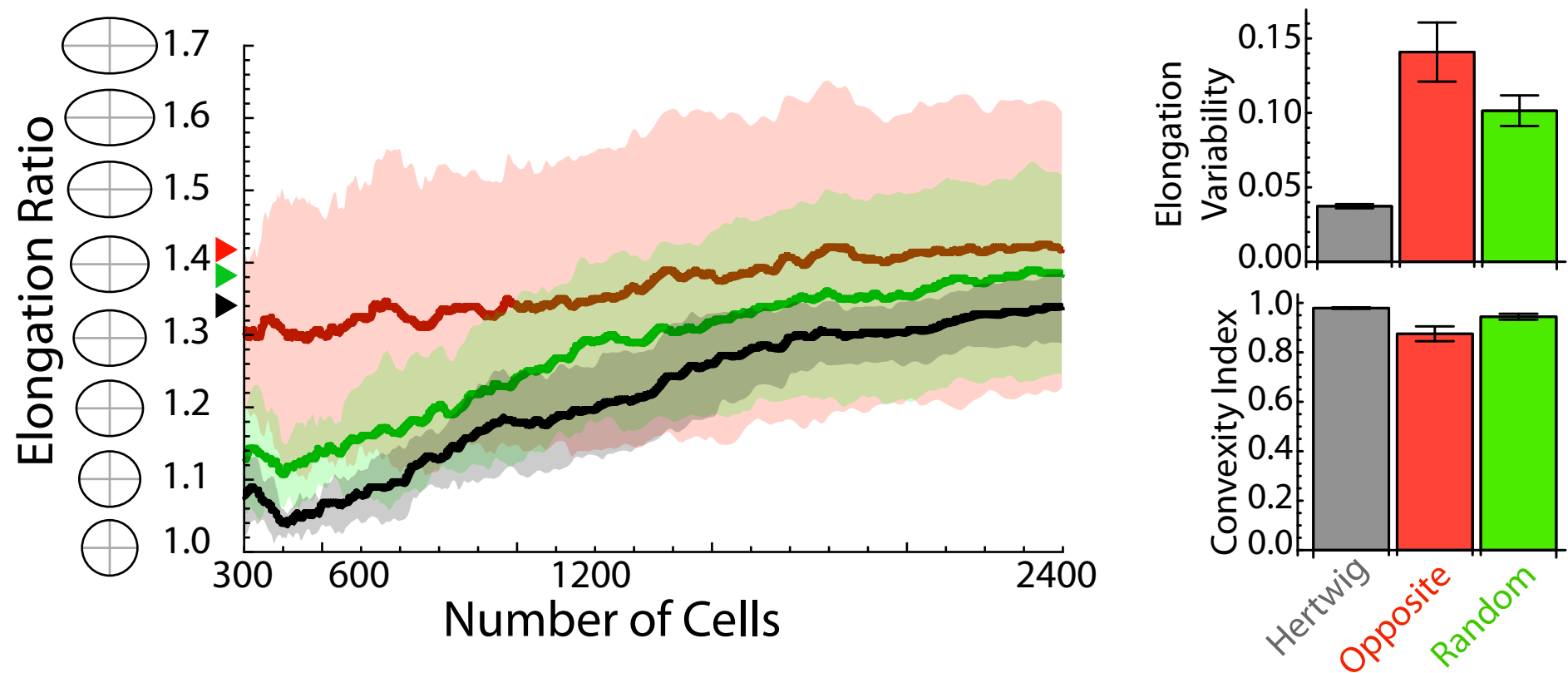

B

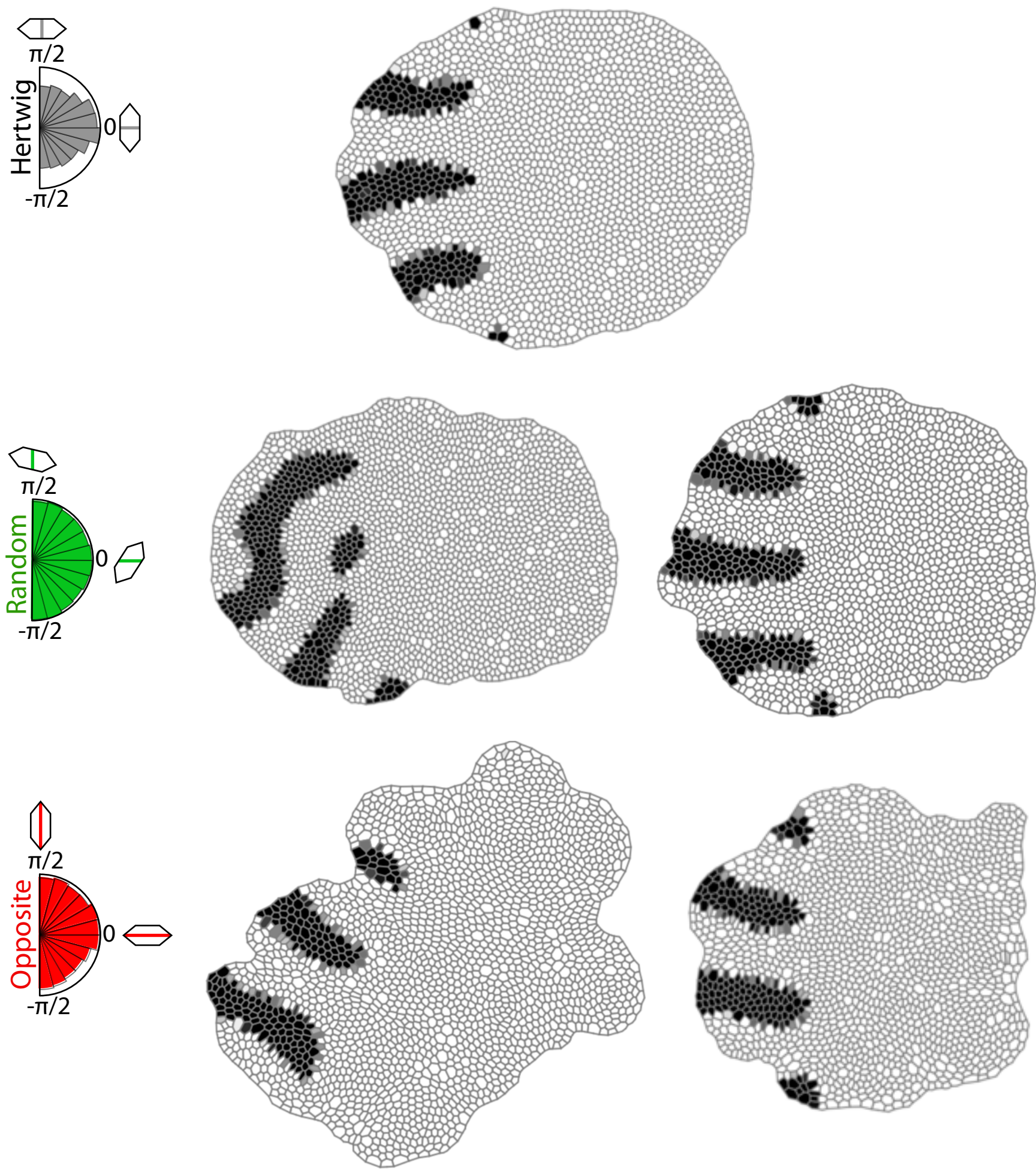

Supplement: Supplementary file 1 — Supplementary file1 (ZIP 366485 kb) [file 41598_2020_67413_MOESM1_ESM.zip › FigS3.pdf]
